# A current view of oncology in Argentina

**DOI:** 10.3332/ecancer.2016.622

**Published:** 2016-02-16

**Authors:** Adrián Pablo Huñis

**Affiliations:** Buenos Aires Oncology Centre, Avenida Hipólito Yrigoyen 4221, Ciudad Autónoma de Buenos Aires C1212ACA, Argentina

**Keywords:** Argentine, oncology, National Cancer Institute (INC)

## Abstract

Since 2010, with the creation of the National Cancer Institute, the Argentine Republic has been tackling the battle against cancer as a genuine public health problem.

Today in Argentina, there is a “cancer policy” whose pillars are prevention, education, assistance, and research.

In this article, we provide information about the incidence and mortality of the tumours most common in adults and children, and details of some epidemiological aspects and advances Argentina has achieved in the battle against cancer in the past decade.

## Introduction

Argentina is a very large country, and there are considerable inequalities between the economic resources of its different regions; these differences are further reflected in the health of their inhabitants.

For more than 30 years of this country’s democratic life, the efforts of different governments have made it possible to reduce part of the difference between the economic, technological, and human resource capacity of each zone; however, regional differences still persist when it comes to access to healthcare in general, and with oncological treatment in particular.

In the words of the former director of the Pan American Health Organisation, Dr Mirta Roses, “We are a federal country, but there are major inequalities between the economic and infrastructure capacity of the provinces, and our system is highly fragmented, divided between the public, the private, and the social sectors. It is difficult to achieve governance, equity, and solidarity owing to this fragmentation” [1].

The health system is actually very complex. It consists of the public sector (national, municipal, and state hospitals), social security, the private sector, and trade unions providing medical care. Depending on the sector, some Argentinians have access to the most advanced technologies, while others must wait hours just for an appointment at a primary healthcare centre.

Despite these difficulties, progress has been made as highlighted by international organisations, such as the reduction in the infantile mortality rate from 16.5 deaths for every 1000 live births in 2003 to 10.8 in 2013 [2]. The mortality rate for heart attacks in public hospitals also fell, from 14.5% in 2005 to 12.8% in 2012.

Other achievements over the past 15 years have included: an increase in the number of organ transplants, which rose 158% in response to active policy in this field [3]; approval of the Sexual Health and Responsible Procreation Law [4], which guarantees free provision of contraception; the Humanised Birth Law [5]; and the National Vaccination Schedule [6], giving free provision of 19 vaccines [6].

## Cancer in the Argentine Republic

The World Health Organisation (WHO) reports that cancer is one of the leading global causes of death. According to 2012 statistics, it caused 8.2 million deaths worldwide and almost 62,000 in Argentina. It is forecast that cancer deaths will continue to increase worldwide, reaching 13.1 million in 2030 [7].

One major advance in Argentina was the creation in 2010 of the National Cancer Institute (INC), which is a department of the National Health Ministry. The creation of the INC places cancer high on the government’s health agenda. Its principal aim is to reduce the incidence of and mortality rate for cancer in Argentina, while also improving the quality of life of people affected by this disease.

The INC is responsible for developing and implementing specific public policies, and for coordinating measures related to information and prevention, early diagnosis, treatment, and rehabilitation. It is also responsible for cancer research in Argentina and for related human resource training.

In addition, its activities include the development of norms for the comprehensive care of patients with cancer, the reduction of risk factors, the training of specialist professionals, and the establishment of a system of epidemiological surveillance and analysis [8].

## Incidence of cancer in Argentina

Argentina has a medium to high incidence of cancer (between 172.3 and 242.9 cases per 100,000 inhabitants) according to the 2015 estimates of the International Agency for Research on Cancer (IARC) [9].

The WHO reports that there are more than 100,000 new cases of cancer per year in both sexes, with similar percentages for both men and women.

**Figure 1.**
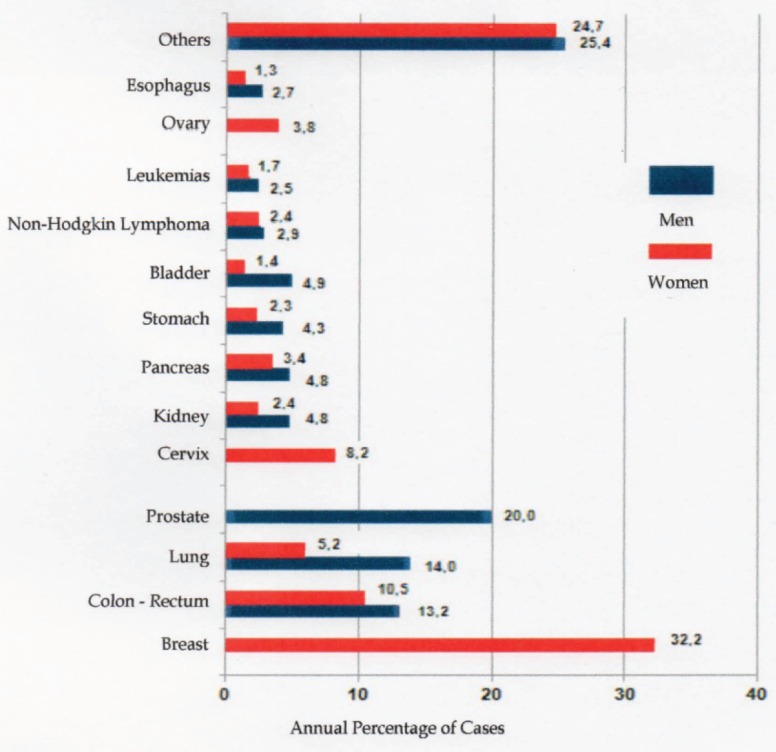
Annual percentage of different tumours in the Argentine Republic, based on 2012 statistics of the INC [10].

**Figure 2.**
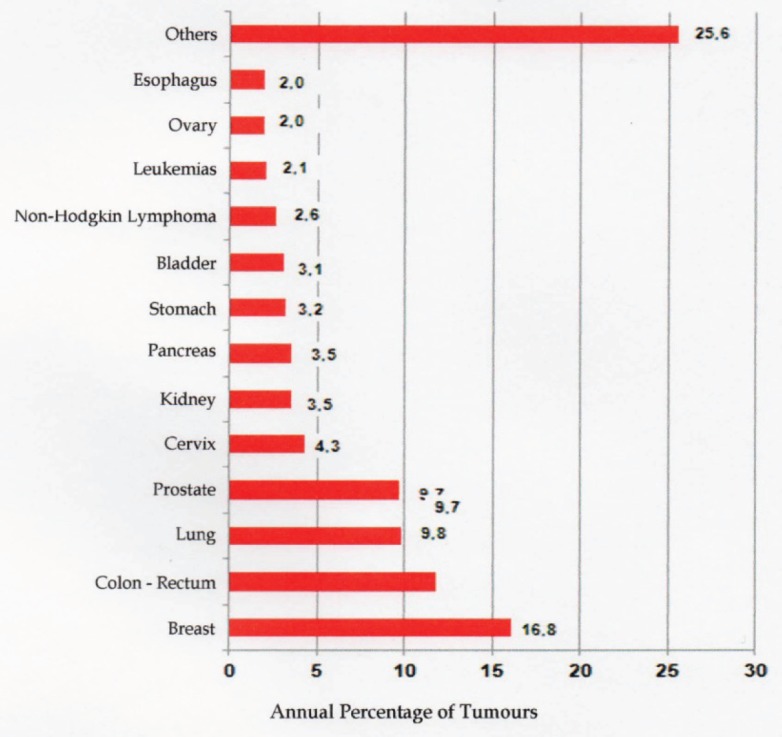
Annual percentage of cancer cases, distributed by sex, based on 2012 statistics of the INC [10].

The most commonly occurring cancers are breast cancer in women, with a rate of 71 cases for every 100,000 women, followed by prostate cancer (44 per 100,000 men), and lung cancer in men (32.5 per 100,000 men). (Figure 1) shows the annual percentage for the different malignant tumours in the Argentine Republic. (Figure 2) shows the distribution of malignant tumours by sex.

## Mortality

The leading cause of death worldwide is non-communicable disease (NCD). In 2008, 57 million people died, and the leading causes were cardiovascular pathologies, cancer, diabetes, and chronic pulmonary conditions.

The second most common cause of NCD death is cancer, which accounted for 7.6 million deaths, most of them in countries with low to medium socioeconomic development.

According to the WHO, many of the NCDs are preventable and share similar risk factors. The leading risk factors–responsible for 30% of cancer deaths–are related to habitual behaviour: a sedentary lifestyle, smoking, high body mass index (BMI), excessive alcohol consumption, and low consumption of fruit and vegetables.

Along with age, the leading risk factor is smoking, which caused 22% of global cancer deaths in general and 71% of deaths from lung cancer. Infections caused by the hepatitis B (HBV) and C (HVC) viruses, or by the human papilloma virus (HPV) may be the cause of malignant tumours that lead up to 20% of cancer deaths in countries with low to medium socioeconomic development [11].

## Mortality in Argentina

According to INC data, NCDs cause more than 60% of all deaths in Argentina each year, 20% of which are because of malignant tumours. A total of 60,000 people per year die of cancer. It is the most frequent cause of death for people aged between 40 and 64 years, the second most frequent for those aged between 5 and 39, and the second most frequent for those aged over 64 years.

Lung cancer is the most common cause of mortality from cancer in all regions except the Cuyo region, where breast cancer is the most common cause. In the other regions, lung cancer is followed by colorectal cancer and breast cancer. The exception are the north-east where prostate cancer is in third place, and the south where stomach cancer is in third place.

## Mortality by gender

In men, lung cancer was responsible for 70% of cancer deaths.

In women, breast cancer is the leading cause of mortality from cancer, with an estimated mortality rate of 18.0 per 100,000 women. In second place comes lung cancer, and in third place is the colorectal cancer. Although the mortality rate for lung cancer has been decreasing for men since 1980, it has been increasing among women owing to the rise in smoking [11].

One characteristic of cervical cancer in north-eastern Argentina is that it is among the five most common causes of cancer deaths, despite the fact that it does not even figure among the ten leading causes of mortality from cancer at the national level [12].

## Cancer in the under-15s

There is a very low incidence of cancer in Argentina among those under the age of 15 years. According to the Argentine Hospital Oncopaediatric Register (ROHA), which is part of the INC, incidence stands at around 1270 cases per year or 124 cases per million–lower than in Germany, Spain, Italy, and the United States, with levels of 132–150 cases per million [13].

In this age group, 30–40% of malignant tumours correspond to leukaemia, 20% to brain tumours, and 13% are lymphomas. Although incidence is lower than in European countries, the survival rate is 65%, compared with levels of 70–80% in more developed countries. This difference is attributable to early diagnosis. In Argentina it is noted that children continue to arrive for initial consultation with advanced tumours, reducing the likelihood of a cure. Moreover, access to the complex diagnostic and therapeutic capacity required for many of these tumours is not available in all regions.

## Prevention

The WHO reports that smoking is the leading avoidable cause of cancer in the world. A 40% of malignant tumours in adults could be prevented through lifestyle changes such as avoiding smoking, a healthy diet, and regular exercise [14].

According to INC data, 4900 new cases of cervical cancer are diagnosed in Argentina each year and 2000 women die of this disease. However, this could easily be prevented through Pap smear tests and adequate treatment. More than 99% of cases are linked to sexually transmitted infections involving the human papilloma virus (HPV) [15].

An interesting survey by the Centre for the Study of the State and Society (CEDES) carried out on 1200 women in the city of Buenos Aires and its suburbs in 2010 found that 85% of them were unaware of the aetiology of cervical cancer and 33% did not know what prevented it.

Cervical cancer, together with maternal mortality, is an indicator that clearly reflects the lack of equity in healthcare. Since it principally affects women of low socioeconomic status i.e. in one of the country’s poorest regions, this disease constitutes a debt of the state vis-à-vis the poorest sectors.

From 2011, the HPV vaccine was incorporated into the National Vaccination Schedule. This vaccine enables girls to be protected against two types of HPV with high-oncogenic risk: genotypes 16 and 18, which are responsible for 77% of cases of cervical cancer. However, side effects have been reported that call into question its suitability [16].

In addition to these preventative measures, a number of tests enable early diagnosis of cancer, thereby making it possible to achieve better therapeutic results with treatment. We refer, for example, to the mammogram for early detection of breast cancer and the colonoscopy for those who have a family history of colon cancer.

## Conclusion

In recent years, the establishment of new hospitals, the incorporation of imaging technology that facilitates early diagnosis, as well as advances in chemotherapy and radiotherapy, have definitely improved cancer figures in Argentina. Nonetheless, an enormous gap persists between different populations based on their socioeconomic status. This in turn has an adverse impact on the adequacy of prevention and early treatment of this disease.
